# Comparative Effects of Snake Envenomation on Rabbit Carcass Decomposition and Insect Succession in a Forensic Context

**DOI:** 10.3390/insects17030274

**Published:** 2026-03-03

**Authors:** Abdelwahab Khalil, Eman E. Zaher, Mustafa M. Soliman, Ashraf M. Ahmed, El-Sayed H. Shaurub, Areej A. Al-Khalaf, Mahmoud M. Zidan

**Affiliations:** 1 Entomology Division, Zoology Department, Faculty of Science, Beni-Suef University, Beni-Suef 62521, Egypt; 2 Department of Zoology, Faculty of Science, Zagazig University, Zagazig 44519, Egypt; 3 Entomology Department, Faculty of Science, Cairo University, Orman, Giza 12613, Egypt; msoliman@cu.edu.eg (M.M.S.);; 4 Zoology Department, Faculty of Science, King Saud University, Riyadh 11481, Saudi Arabia; 5 Biology Department, College of Science, Princess Nourah bint Abdulrahman University, Riyadh 11671, Saudi Arabia; 6 Zoology & Entomology Department, Faculty of Sciences, Al-Azhar University, Cairo 11884, Egypt

**Keywords:** forensic entomology, snake venom, *Naja haje*, *Cerastes cerastes*, postmortem interval, decomposition, arthropod succession, Diptera, Coleoptera, Hymenoptera

## Abstract

Background: Snake envenomation represents a significant health concern in some regions of the world, with fatal cases occasionally requiring forensic investigation to estimate the postmortem interval (PMI). However, the influence of venom on carrion decomposition dynamics and arthropod succession patterns remains poorly understood, potentially compromising postmortem interval (PMI) estimations in such cases. Objectives: This study investigated the effects of *Naja haje* and *Cerastes cerastes* venoms on decomposition progression and necrophagous arthropod succession. Methods: Fifteen rabbits were allocated into three experimental groups. Two groups received median lethal intravenous doses (LD_50_) of *N. haje* or *C. cerastes* venom, whereas the control group received a saline injection followed by CO_2_ euthanasia. The carcasses were subsequently placed under natural field conditions and monitored daily for 15 days. Results: The presence of venom significantly altered decomposition dynamics. *C. cerastes* venom accelerated early decomposition, shortening both the fresh stage (1 ± 0.22 days vs. 2 ± 0.31 days in controls,) and bloating stage (3 ± 0.35 days vs. 5 ± 0.35 days), while extending both the decay stage (6 ± 0.3 days vs. 6 ± 0.17 days) and the dried stage (5.0 ± 0.44 days vs. 2 ± 0.039 days). *N. haje* venom showed intermediate effects. Overall arthropod abundance peaked on day 5 and declined thereafter. Control carcasses exhibited significantly higher arthropod abundance than carcasses envenomed with *C. cerastes* or *N. haje*. Conclusions: Snake envenomation significantly influenced decomposition kinetics and arthropod colonization patterns. Envenomation with *C*. *cerastes* venom produced more pronounced alterations than envenomation with *N. haje* venom.

## Simple Summary

This study investigates the effects of envenomation with venom from the horned viper (*Cerastes cerastes*) and the Egyptian cobra (*Naja haje*) on rabbit carcass decomposition and the succession of necrophagous arthropods, a critical component of forensic entomology. Compared with the control group, snake envenomation shortened the fresh and bloated stages of decomposition and reduced overall arthropod abundance. Notably, *C. cerastes* venom produced more significant alterations than *N. haje* venom. The findings demonstrate that snake venom significantly influences insect succession patterns and carcass decomposition dynamics. These results indicate that postmortem interval (PMI) estimations in snake envenomation cases may require venom-specific adjustments to standard forensic entomological models.

## 1. Introduction

As a branch of forensic entomology, medico-legal entomology examines insects and other arthropods associated with legal investigations [1]. Most significantly, insects have been demonstrated to serve as reliable tools in forensic entomology for estimating the minimum postmortem interval (mPMI), i.e., the duration between the moment of death and the discovery of the corpse [2]. Forensic entomologists precisely calculate the moment of oviposition, then based on this information and taking into account biotic factors, including species identification, and abiotic factors, including climate, latitude, and geographic location, the mPMI can be estimated [3]. Blowflies (Diptera: Calliphoridae) and flesh flies (Diptera: Sarcophagidae) are cosmopolitan in distribution and exhibit high reproductive rates on carrion. Their feeding and breeding behavior renders them significant in medical, veterinary, and forensic contexts [4]. They are usually the first of the faunal succession to arrive on human cadavers and are the primary and most accurate forensic indicators of the time of death [5]. Following flies, different orders of insects (Coleoptera, Hymenoptera, and Lepidoptera) come as decomposition progresses [6].

Chemical compounds present in a corpse, including medications, insecticides, or poisons, can alter insect activity, succession, and decomposition processes, potentially leading to inaccurate postmortem interval (PMI) estimations. Therefore, detecting drugs and other toxic substances at various stages of decomposition can improve the accuracy of PMI estimation and facilitate the determination of the cause of death in decomposed remains, providing more precise forensic evidence [7,8].

While not among the leading global causes of mortality, envenomation by venomous animals represents a significant yet neglected public health challenge, particularly in tropical and subtropical regions [9,10,11]. The World Health Organization (WHO) estimates that millions of people, primarily in Africa, the Middle East, and Asia, are afflicted by snakebites annually [12,13,14]. Although snakebite fatalities—estimated at 81,000 to 138,000 deaths globally per year—are fewer than those caused by major infectious or non-communicable diseases, they represent a critical category of accidental and occasionally suspicious deaths that require specialized forensic attention [15]. In countries like Egypt, where approximately 200 fatalities occur annually from poisonous animals, these cases often present significant diagnostic challenges during forensic investigations, particularly when the body is in an advanced state of decomposition [15,16]. Despite these findings may suggest the nonexistence of death cases due to envenomation by venomous animals in certain countries, they highlight the importance of envenomation from the standpoint of forensic entomology.

Diagnosing fatal snakebite through internal anatomical examination during autopsy remains challenging [11]. Consequently, forensic investigations typically rely on clinical manifestations to infer the species of biting snake [16]. Despite increased research on synthetic chemicals, pharmaceuticals, and snake venoms [17,18,19,20,21,22,23,24], limited information exists regarding the effects of physiologically active toxins, particularly snake venoms, on carrion decomposition and arthropod succession. This study aimed to investigate the effects of Egyptian cobra (*Naja haje*) and horned viper (*Cerastes cerastes*) venoms on decomposition progression and necrophagous arthropod succession using rabbit carcasses as experimental models. The findings provide valuable insights for distinguishing mortality caused by venomous animals and for improving postmortem interval (PMI) estimations in forensic investigations.

## 2. Materials and Methods

### 2.1. Study Site and Meteorological Monitoring

**Figure 1:**
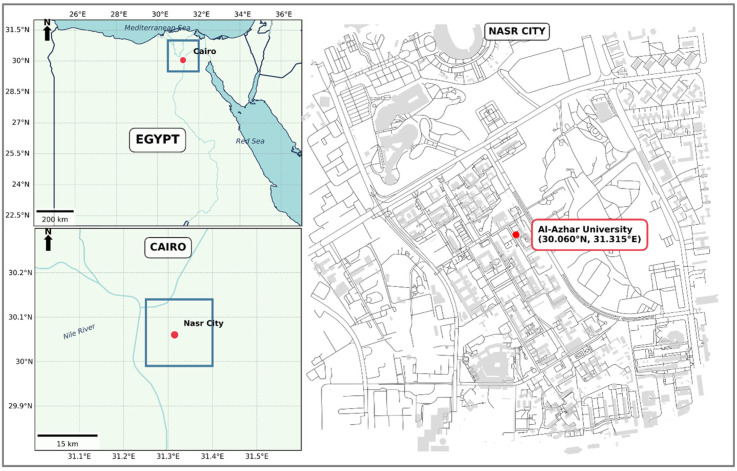
Maps illustrating the geographical location of the study site.

This investigation was conducted from 2–17 August 2022, at the College of Science Garden, Al-Azhar University, Nasr City, Cairo, Egypt (30.060055° N, 31.314697° E) (Figure 1). Daily meteorological measurements, including relative humidity (%), ambient temperature (°C), and wind speed (km/h), carcass surface temperature (°C), and soil temperature (°C), were recorded throughout the experimental period following a standardized protocol [25] as follows: the ambient weather temperature and carcass surface temperature, and relative humidity were measured daily by calibrated digital thermometer and hygrometer devices (Elitch, Jiangsu, China), respectively, according to the instruction manual. Wind speed was daily monitored using Skywatch Wind Meter device (Skywatch, JDC Electronic, Yverdon-les-Bains, Switzerland) according to the instruction manual. All environmental data and carcass surface temperature were collected at consistent time points each day, particularly during early morning hours, to ensure comparability across the study period. Carcass temperature was obtained from the external abdominal surface of each carcass under shaded conditions to avoid direct solar exposure. Therefore, recorded surface temperatures represent external carcass conditions rather than internal postmortem heat.

### 2.2. Experimental Animals and Venom Preparation

Due to the limited availability of larger animal models comparable to humans, rabbits were used as the experimental model. Fifteen European domestic rabbits (*Oryctolagus cuniculus domesticus*; Lagomorpha, Leporidae), each weighing approximately 2.5 kg (2–3 kg), were obtained from the Egyptian Holding Company for Biological Products and Vaccines (VACSERA) Animal House (https://www.vacsera.com/#home, accessed on 28 February 2026). This model is consistent with previous forensic decomposition studies [26,27]. All animals were handled carefully to avoid external injuries that could affect arthropod colonization patterns and succession dynamics. The experimental protocol was reviewed and approved by the Research Animal Facility—Institutional Animal Care and Use Committee (URAF-IACUC), Cairo, Egypt (Approval No.18-22).

Snake venoms were obtained from two medically significant native Egyptian species: the Egyptian cobra (*N. haje*), recognized as one of the most venomous snakes in Africa, and the horned viper (*C. cerastes*), which inhabits the desert regions of North Africa. Venom samples were generously provided by the Africano Tolba Zoo Company, Giza, Egypt. Prior to experimental use, the venoms were reconstituted in 0.85% physiological saline (pH 7.2) to achieve a stock concentration of 1 mg/mL (*w/v*) [28] as adapted [25].

### 2.3. Determination of Median Lethal Dose (LD_50_)

The median lethal dose (LD_50_) for both *N. haje* and *C. cerastes* venoms was determined [25,29]. These preliminary toxicological assessments established the dose–response relationships required to calculate the experimental LD_50s_ applied in this study.

### 2.4. Experimental Design and Envenomation Protocol

Rabbits were randomly assigned to three treatment groups (*n* = 5 per group). The first group received intravenous injections of *N. haje* venom at the calculated LD_50_ dose (12.8 μg/g body weight). The second group received intravenous injections of *C. cerastes* venom at its LD_50_ dose of (62.0 μg/g body weight) [25]. The third group served as the control and received intravenous injections of 0.85% physiological saline, followed by euthanasia via carbon dioxide (CO_2_) asphyxiation [30].

Following venom administration, envenomed rabbits were continuously monitored in their cages until death was confirmed. Death occurred approximately 10 min post-injection for *N. haje* and 20 min post-injection for *C. cerastes*. Upon confirmation of death, all carcasses were immediately transported to the experimental field site within one hour to minimize any artificial delays in the natural colonization process. The experimental design and field setup were adapted from our previous work [25].

### 2.5. Insect Collection and Identification

Throughout the 15-day observation period, adult arthropods from three orders—Diptera (flies), Coleoptera (beetles), and Hymenoptera (ants)—were collected daily from each carcass. Adult flying insects visiting the carcasses and both crawling adults and larvae were collected using a sweep net and spatula, respectively. Collection was carried out twice daily, in the early morning and evening hours. Specimens were immediately preserved in labeled vials containing 70% ethanol. Each vial was marked with the collection date, carcass identification number, and treatment group. Moreover, immature stages, including eggs and larvae were collected and reared in the laboratory till metamorphosing to adult stages where they were included in the data. All collected specimens were subsequently identified to the species level. All insects were enumerated to assess daily abundance patterns for each order across treatment groups and decomposition stages.

### 2.6. Decomposition Stage Assessment

Carcass decomposition was monitored daily and classified into four distinct stages according to established forensic taphonomic criteria: (1) Fresh stage—characterized by minimal physical changes, absence of bloating, and initial insect colonization; (2) Bloated stage—marked by visible abdominal distension due to accumulated decomposition gases, skin discoloration, and peak dipteran activity; (3) Decayed stage—defined by the collapse of body cavities, extensive tissue liquefaction, active maggot feeding, and progressive loss of soft tissue; and (4) Dried stage—characterized by desiccation of remaining tissues, mummification, and predominance of late-arriving scavenger species. The duration of each decomposition stage was recorded for each carcass to assess treatment-specific effects on decomposition dynamics.

### 2.7. Statistical Analysis

The duration of each decomposition stage (fresh, bloated, decayed, and dried) was compared among treatment groups using the non-parametric Kruskal–Wallis H-test. This approach was selected because the small sample size (n=5 per group) precludes robust testing for normality (e.g., Shapiro–Wilk test) and increases the risk of violating parametric assumptions. Post hoc pairwise comparisons were conducted using Dunn’s test with the Bonferroni correction for multiple comparisons. Statistical significance was set at α = 0.05. Results are presented as mean ± standard deviation (SD) to facilitate comparison with existing forensic entomology literature.

Given the count nature of the insect abundance data and the presence of overdispersion (variance exceeding the mean), negative binomial regression was used to model the relationships between abundance (response variable) and predictor variables, including day (continuous), treatment group (categorical: control, *N. haje*, *C. cerastes*), decomposition stage (categorical: fresh, bloated, decayed, dried), and insect order (categorical: Coleoptera, Diptera, Hymenoptera). The negative binomial distribution accounts for over-dispersed count data more appropriately than Poisson regression by incorporating an additional dispersion parameter (θ).

Model fit was evaluated using the Akaike Information Criterion (AIC), deviance statistics, and examination of residuals. Analysis of deviance, employing Type II likelihood ratio tests, was conducted to assess the overall significance of each predictor variable. Pairwise comparisons were performed using Tukey’s method to control for family-wise error rates. All parameter estimates are reported on the log scale, including standard errors, z-values, and *p*-values.

Spearman rank correlation coefficients were calculated to assess non-parametric associations between arthropod order abundances and environmental variables, including ambient temperature, carcass temperature, and relative humidity. Spearman’s method was selected because it is robust to non-normal distributions and capable of detecting monotonic relationships. Correlation matrices were visualized as heatmaps, with color gradients representing both the strength and direction of the correlations.

All statistical analyses were conducted using R 4.3.2 statistical software (R Core Team, 2023) [31]. Negative binomial regression analyses were conducted with the MASS package [32]. Data visualization was produced using ggplot2 [33], while data management and manipulation were facilitated by the dplyr and tidyr packages [34]. Statistical significance was defined as *p* < 0.05 for all tests, unless stated otherwise.

## 3. Results

### 3.1. Meteorological Conditions

**Figure 2:**
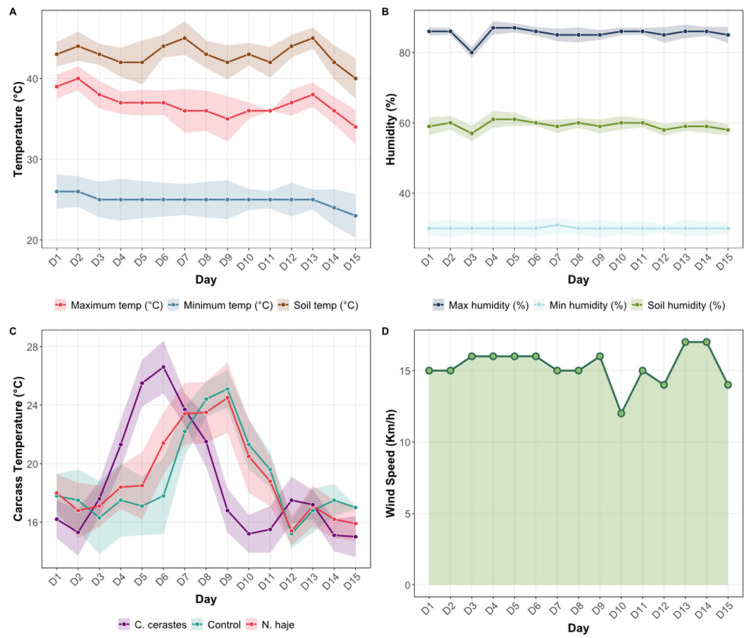
Mean meteorological conditions of the surrounding environment and carcasses at the experimental site over the 15-day study period. Daily measurements of (**A**) ambient temperature (maximum and minimum air temperature, and soil temperature, °C), (**B**) relative humidity (maximum and minimum air humidity, and soil humidity, %), (**C**) carcass surface temperature (°C) across the three experimental groups (*Cerastes cerastes* envenomation, *Naja haje* envenomation, and control), and (**D**) wind speed (km/h) over 15 days (D1–D15). Shaded areas in panels A–C represent standard deviation.

Environmental conditions during the 15-day experimental period are presented in Figure 2. Ambient temperatures exhibited substantial daily variation, with maximum temperatures ranging from 43 °C to 48 °C (Mean ± SD: 45.2 ± 1.8 °C) and minimum temperatures fluctuating between 22 °C and 26 °C (Mean ± SD: 23.7 ± 1.2 °C) throughout the study period (Figure 2A). Soil temperature remained relatively stable, ranging consistently between 36 °C and 40 °C (Mean ± SD: 38.1 ± 1.1 °C) across all observation days.

Relative humidity measurements indicated minimal variation during the experimental period (Figure 2B). Maximum relative humidity remained consistently high, ranging from 82% to 88% (Mean ± SD: 84.3 ± 1.9%), whereas minimum relative humidity was more stable, maintaining values between 58% and 62% (Mean ± SD: 60.1 ± 1.3%). Soil humidity exhibited the least variability among the measured parameters, remaining nearly constant at approximately 28–30% (Mean ± SD: 29.2 ± 0.7%) throughout the 15-day study period.

Carcass surface temperatures varied according to treatment group and progression through decomposition stages (Figure 2C). The *C. cerastes* venom group exhibited the highest temperature peaks, reaching approximately 27 °C between days 5 and 7. In contrast, the control and *N. haje* venom groups displayed similar but slightly lower temperature profiles during the same period. By day 10, all three groups converged to comparable temperature ranges, between 17 °C and 19 °C, as decomposition progressed into the later stages.

Wind speed remained moderate throughout the experimental period, ranging from 10 to 17 km/h (Mean ± SD: 14.2 ± 2.1 km/h), with peak values recorded on days 13–14 (Figure 2D). These relatively stable meteorological conditions provided a consistent environmental context for assessing the effects of snake venom on carcass decomposition dynamics and insect succession patterns.

### 3.2. Decomposition Stage Progression

**Figure 3:**
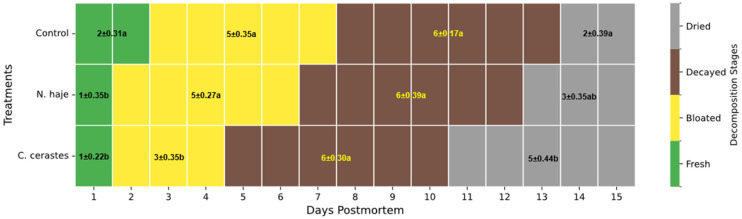
Comparative timeline of decomposition stages in rabbit carcasses following envenomation by *Naja haje* and *Cerastes cerastes* venoms. Values are presented as mean duration ± standard deviation over the 15-day study period. Unlike lowercase letters vertically within the same color denote significant differences (Kruskal–Wallis H-test followed by Dunn’s post hoc test, *p* < 0.05).

The presence of snake venom significantly influenced both the progression and duration of the four decomposition stages compared with the control group (Figure 3). Overall, envenomation, particularly by *C. cerastes*, accelerated transitions between stages, resulting in a faster overall decomposition process. Envenomation notably reduced the duration of the fresh stage. Control carcasses remained in the fresh stage for 2 ± 0.31 days, whereas carcasses exposed to *N. haje* and *C. cerastes* progressed to the bloated stage earlier, remaining in the fresh stage for only 1 ± 0.35 days and 1 ± 0.22 days, respectively. These differences were statistically significant (H = 9.23, *p* < 0.05). The bloated stage also varied among treatments. The control and *N. haje* venom-treated carcasses exhibited similar durations of 5 ± 0.35 and 5 ± 0.27 days, respectively, whereas the *C. cerastes* venom-treated group showed a notably shorter bloated stage of 3 ± 0.35 days (H = 10.10, *p* < 0.05). The duration of the decayed stage remained consistent across all groups, lasting 6 days in each case (Control: 6 ± 0.17 days; *N. haje*: 6 ± 0.39 days; *C. cerastes*: 6 ± 0.30 days). Although the total decomposition duration did not differ significantly (H = 0.049, *p* > 0.05), envenomed carcasses entered the decayed stage earlier due to accelerated progression through the fresh and bloated stages. By the end of the 15-day study period, distinct differences were observed in the duration of the dried stage. *C. cerastes* venom-treated carcasses remained in this final stage for 5 ± 0.44 days, significantly longer than the control group, which remained for only 2 ± 0.39 days (H = 12.29, *p* < 0.05). The *N. haje* venom-treated group exhibited an intermediate duration of 3 ± 0.35 days, which was not significantly different from either the control or the *C. cerastes* venom-treated group.

### 3.3. Necrophagous Insects’ Abundance

**Figure 4:**
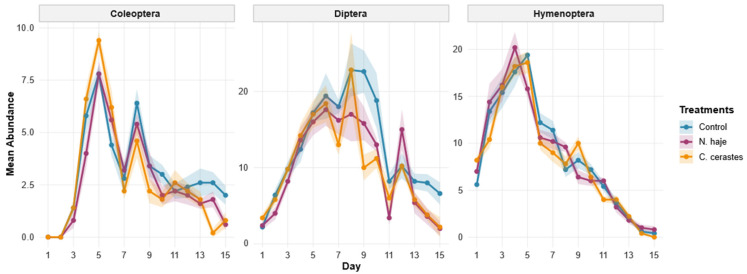
Temporal patterns of insect abundance, categorized by order (Coleoptera, Diptera, and Hymenoptera), were recorded on rabbit carcasses over a 15-day experimental period. Comparisons were made between control carcasses and those treated with *Naja haje* and *Cerastes cerastes* venoms. Data points represent the mean ± standard error (SE) from five replicate trials, each involving a distinct rabbit per venom treatment.

A total of 675 observations were recorded across all treatment groups, decomposition stages, and insect orders over the 15-day study period. Overall insect abundance showed considerable variation (Mean ± SD: 7.49 ± 6.56 individuals per observation; range: 0–31), with a median of 6.00 individuals. A total of 30 species were identified across three orders: 21 dipteran species belonging to 10 families (Calliphoridae, Muscidae, Sarcophagidae, Drosophilidae, Fanniidae, Hippoboscidae, Phoridae, Piophilidae, Sphaeroceridae, and Ulidiidae), six coleopteran species from four families (Cleridae, Dermestidae, Staphylinidae, and Tenebrionidae), and three hymenopteran species within the family Formicidae (Table 1). Colonization patterns of the three primary insect orders—Coleoptera, Diptera, and Hymenoptera—displayed distinct temporal successions throughout the study, with differences in abundance observed between the control and venom-treated groups. (Figure 4). Coleoptera exhibited delayed colonization, with negligible presence until day 3. All groups reached a primary peak on day 5. The *C. cerastes* venom-treated group recorded the highest mean abundance (9.4 ± 0.2), followed by the *N. haje* venom-treated group (7.8 ± 0.4) and the control group (7.5 ± 0.3). After a brief decline, a secondary peak occurred on day 8, during which the control group (6.4 ± 0.5) maintained higher abundance than the *N. haje* (5.4 ± 0.3) and *C. cerastes* (4.6 ± 0.2) venom-treated groups. During the final decomposition stages (days 13–15), beetle abundance remained significantly higher in control carcasses (2.6 ± 0.4 on day 13), whereas both venom-treated groups declined to near-zero levels. The dominant coleopteran species were *Dermestes maculatus* (Dermestidae), recorded across all treatments and decomposition stages, and *Necrobia rufipes* (Cleridae), both well-established forensic indicators of the active decay and dry stages (Table 1).

Diptera abundance peaked sharply on day 1 and displayed complex fluctuations throughout the experiment. The control and *C. cerastes* venom-treated groups reached their maximum abundance on day 8, with mean values of 23.5 ± 3.4 and 23.1 ± 2.1, respectively. In contrast, the *N. haje* venom-treated group reached its primary peak earlier, on day 6 (17.5 ± 1.8), followed by a lower secondary peak on day 8 (17.0 ± 1.5). A notable late-stage increase occurred in the *N. haje* group on day 12 (14.5 ± 2.6), a pattern not observed in the other treatments. By day 15, the control group maintained a higher mean abundance (7.1 ± 1.2) than both the *N. haje* (2.3 ± 0.6) and *C. cerastes* (1.9 ± 0.4) groups. Within Diptera, Calliphoridae was the most speciose family, represented by six species: *Chrysomya albiceps*, *Chrysomya megacephala*, *Chrysomya rufifacies*, *Lucilia cuprina*, *Lucilia sericata*, and *Calliphora vicina*. *C. albiceps* and *L. sericata* were the most broadly distributed calliphorids, recorded across all treatments and all four decomposition stages. Muscidae contributed three species (*Musca domestica*, *Musca sorbens*, and *Musca stabulans*), and Sarcophagidae was represented by *Sarcophaga argyrostoma*, *Sarcophaga dux*, and *Wohlfahrtia nuba*. Five additional families—Drosophilidae (*Drosophila repleta*), Fanniidae (*Fannia canicularis*), Hippoboscidae (*Hippobosca equina*), Phoridae (*Megaselia scalaris*), and Piophilidae (*Piophila casei*)—were recorded primarily during the decay and dry stages, consistent with their known late-successional ecology. Full species distributions across treatments and decomposition stages are detailed in Table 1. Hymenoptera were the earliest order to reach high abundance, peaking between days 4 and 5. The *N. haje* group exhibited the highest overall mean abundance on day 4 (20.2 ± 1.4), while the control and *C. cerastes* groups peaked on day 5 with mean abundances of 19.5 ± 0.5 and 18.6 ± 0.8, respectively. Following these peaks, Hymenoptera abundance declined steadily in all groups, reaching near-zero values by day 15 (Control: 0.4 ± 0.1; *N. haje*: 0.8 ± 0.2; *C. cerastes*: 0.1 ± 0.1). All three Hymenoptera species belonged to the family Formicidae: *Camponotus* sp., *Cataglyphis* sp. and *Messor abeillei*. All three species were consistently present across the fresh, bloated, and decay stages under all three treatments, with activity declining sharply in the dry stage, particularly in the *C. cerastes*-treated group (Table 1).

**Figure 5:**
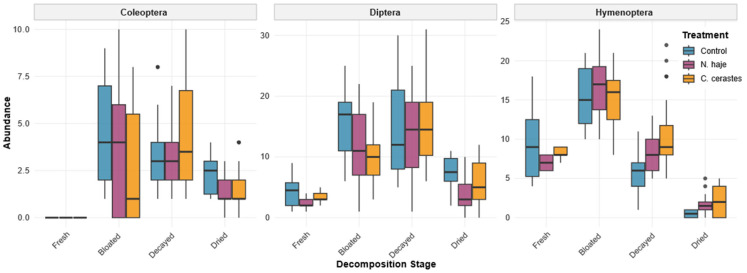
Insect abundance across decomposition stages, venom treatments, and taxonomic orders on rabbit carcasses. Box plots illustrate the distribution of arthropod abundance (Coleoptera, Diptera, and Hymenoptera) across four decomposition stages (fresh, bloated, decayed, and dried) under three treatment conditions (control, *Naja haje*, and *Cerastes cerastes*). Boxes represent the interquartile range (IQR; 25th–75th percentiles), with horizontal lines indicating medians. Whiskers extend to 1.5× IQR, and individual points denote outliers. Sample sizes included 675 observations collected from 15 rabbit carcasses, monitored daily throughout the experimental period.

The abundance of necrophagous insects varied significantly across the four decomposition stages, exhibiting distinct successional patterns for each insect order and treatment group (Figure 5). During the initial fresh stage, insect activity was primarily dominated by Hymenoptera and Diptera, whereas Coleoptera were absent in all treatments. Hymenoptera were the most abundant early colonizers, with the control group showing the highest mean abundance (9.50 ± 1.50), followed by the *C. cerastes* (8.20 ± 0.37) and *N. haje* (7.00 ± 0.45) treatment groups. Diptera displayed a similar pattern, with the highest initial recruitment in the control group (4.30 ± 0.82) compared to the *C. cerastes* (3.40 ± 0.51) and *N. haje* (2.40 ± 0.51) treatments. The transition to the bloated stage was marked by a substantial increase in insect abundance across all orders. Hymenoptera reached their overall peak during this stage, particularly in the *N. haje* treatment group, which recorded the highest mean abundance (16.65 ± 0.89). Diptera populations also increased sharply, with the control group (15.36 ± 1.02) exhibiting higher mean abundance than the *N. haje* (11.88 ± 1.21) and *C. cerastes* (9.93 ± 1.08) treatment groups. Coleoptera began colonizing the carcasses during this stage, with the highest mean abundance observed in the control group (4.44 ± 0.52).

During the decay stage, Diptera emerged as the dominant insect order. The *C. cerastes* treatment group exhibited the highest mean abundance of both Diptera (15.40 ± 1.27) and Coleoptera (4.40 ± 0.55) at this stage. In contrast, Hymenoptera numbers declined across all treatments, although they remained significantly higher in the *C. cerastes* group (10.30 ± 0.80) compared to the control group (5.60 ± 0.47). The *N. haje* group demonstrated intermediate abundance levels for all three orders during active decay. In the final dry stage, overall insect abundance decreased markedly. The control group retained a relatively higher population of Diptera (7.30 ± 0.91) and Coleoptera (2.30 ± 0.33) compared with the envenomed carcasses. Within the venom-treated groups, Diptera abundance declined to 5.60 ± 0.72 in the *C. cerastes* group and 3.67 ± 0.72 in the *N. haje* group. Hymenoptera presence was minimal across all groups, approaching near-zero levels in the control group (0.50 ± 0.17), while small numbers persisted on the envenomed carcasses (*C. cerastes*: 2.12 ± 0.37; *N. haje*: 1.70 ± 0.29).

Overall, the data suggest that envenomation influences both the timing and magnitude of insect colonization. Control carcasses generally supported higher initial and terminal insect populations. In contrast, *C. cerastes* venom appeared to enhance Diptera and Coleoptera recruitment during the active decay stage. *N. haje* venom was associated with the highest peak of Hymenoptera during the bloated stage but tended to result in lower Diptera counts throughout the later stages of decomposition. Complete species-level distributions across all decomposition stages and treatment groups are presented in Table 1.

### 3.4. Statistical Modeling of Abundance Patterns

**Figure 6:**
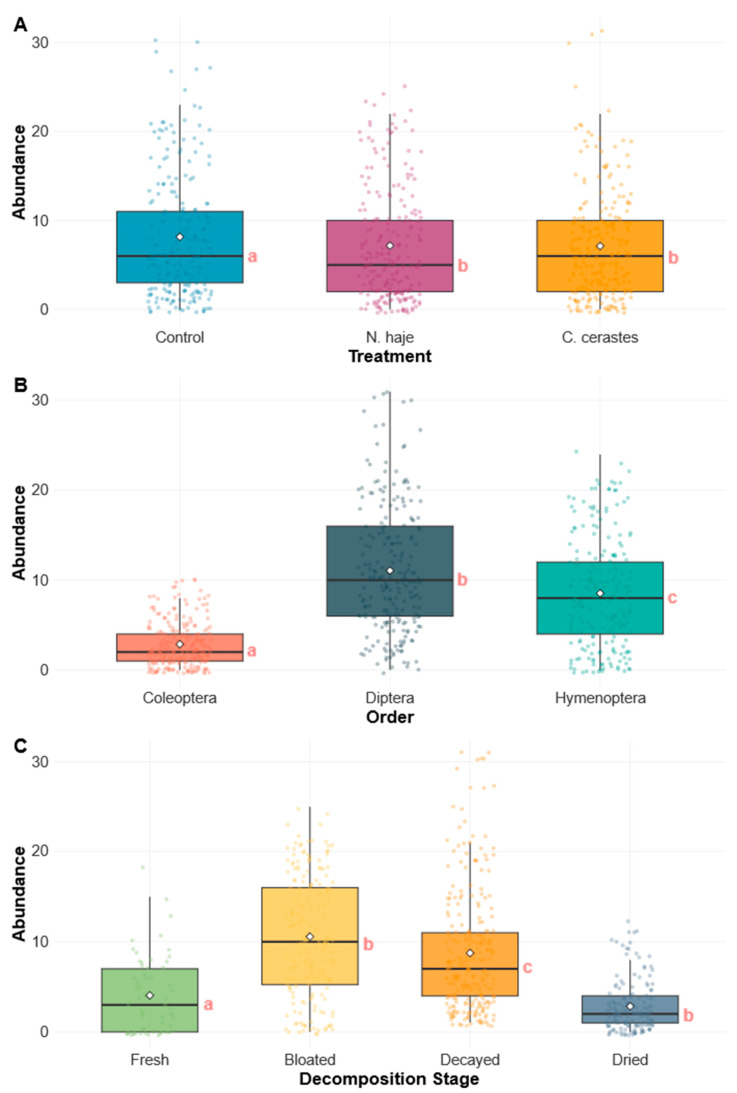
Distribution of insect abundance across experimental factors is illustrated using boxplots with overlaid individual observations. (**A**) Abundance by treatment (control, *Naja haje*, and *Cerastes cerastes*). (**B**) Abundance by insect order (Coleoptera, Diptera, and Hymenoptera). (**C**) Abundance across decomposition stages (fresh, bloated, decayed, and dried). Boxes represent interquartile ranges, with medians indicated by horizontal lines and means shown as points. Whiskers extend to 1.5 × the interquartile range. Each box summarizes five replicates (*n* = 5), corresponding to five individual rabbit carcasses per treatment. Different lowercase letters indicate statistically significant differences among groups, as determined by generalized linear mixed models (GLMMs) with Tukey-adjusted post hoc comparisons (*p* < 0.05).

Negative binomial regression analysis indicated that arthropod abundance was significantly influenced by all predictor variables examined (Table 2). The model demonstrated an excellent fit to the data (θ = 5.52 ± 0.60; AIC = 3542.4), with a substantial reduction in deviance compared to the null model (Null deviance = 1821.96; Residual deviance = 779.39). The day of observation had a significant negative effect on arthropod abundance (β = −0.105, *p* < 0.001), corresponding to an average decline of approximately 9.9% per day over the study period (LR χ^2^ = 43.28, df = 1, *p* < 0.001). Overall treatment effects were statistically significant (LR χ^2^ = 15.15, df = 2, *p* < 0.001). Pairwise comparisons revealed that control carcasses had significantly higher arthropod abundance than those envenomed by *C. cerastes* (β = 0.206, *p* = 0.002). No significant difference was observed between the two venom treatment groups (β = 0.009, *p* = 0.987) (Figure 6A). Insect order had the largest effect size in the model (LR χ^2^ = 537.62, df = 2, *p* < 0.001). Compared to Coleoptera (baseline), Diptera exhibited substantially higher abundance (β = 1.371, *p* < 0.001), corresponding to an approximate 294% increase in numbers. Hymenoptera also showed significantly elevated abundance relative to Coleoptera (β = 1.055, *p* < 0.001), representing an approximate 187% increase. Diptera abundance was significantly higher than that of Hymenoptera (*p* < 0.001), confirming Diptera as the dominant arthropod order throughout the decomposition process (Figure 6B). The decomposition stage had a significant effect on arthropod abundance (LR χ^2^ = 307.04, df = 3, *p* < 0.001). Compared with the bloated stage (baseline), the fresh stage exhibited a pronounced reduction in abundance (β = −1.333, *p* < 0.001), corresponding to approximately 74% fewer arthropods. The decayed stage showed a significant increase in abundance relative to the bloated stage (β = 0.275, *p* = 0.004), whereas the dried stage demonstrated a decrease in abundance (β = −0.336, *p* = 0.051). Pairwise comparisons revealed significant differences between all decomposition stages (all *p* < 0.05), except for the comparison between the bloated and dried stages (*p* = 0.205) (Figure 6C).

These results indicate that insect abundance patterns during carrion decomposition are influenced by complex interactions among temporal dynamics, venom treatment, decomposition stage progression, and taxon-specific colonization strategies. The presence of snake venom, particularly from *N. haje* and *C. cerastes*, led to a significant reduction in overall arthropod abundance compared with control carcasses. These findings have important implications for PMI estimation in forensic investigations involving envenomation.

### 3.5. Temporal Succession Patterns and Abundance-Sequence Dynamics

**Figure 7:**
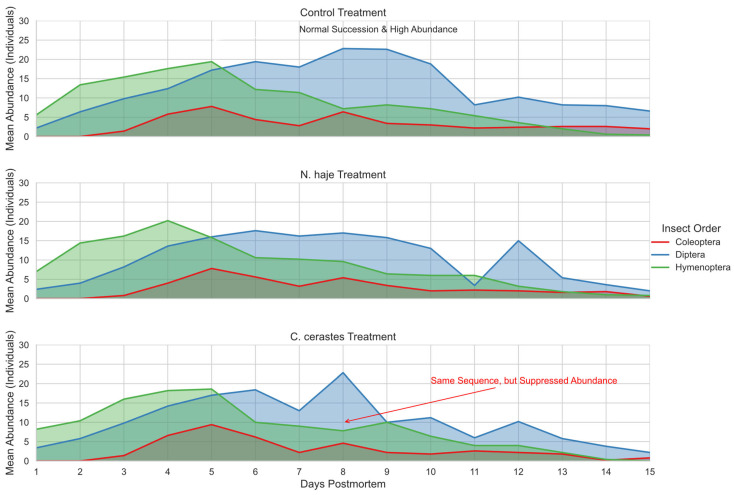
Comparative visualization of arthropod succession waves and abundance dynamics among treatments. The area plots illustrate the mean abundance of the three primary insect orders—Diptera (blue), Hymenoptera (green), and Coleoptera (red)—over the 15-day experimental period for control, *Naja haje*, and *Cerastes cerastes* groups. This conceptual comparison demonstrates that while the fundamental successional sequence (the order of arrival and peak activity) remains largely conserved across all treatments, snake envenomation significantly suppresses peak abundance and alters the temporal kinetics (width and height of the waves), particularly in the *C. cerastes* group.

The temporal succession of arthropod orders exhibited a consistent successional blueprint across all treatment groups, though significant deviations were observed in abundance and timing kinetics (Figure 7). In all carcasses, Hymenoptera and Diptera were the initial colonizers, followed by the gradual recruitment of Coleoptera. As illustrated in the area-density plots (Figure 7), the “succession waves” in the control carcasses were characterized by high peaks and broad temporal overlap. Diptera and Hymenoptera reached their maximum abundance between days 4 and 8, followed by a distinct Coleopteran wave peaking around day 5. In contrast, envenomed carcasses—particularly those treated with *C. cerastes* venom—exhibited a “suppression effect.” While the successional sequence (the chronological order of taxonomic appearance) remained unchanged, the overall abundance was markedly reduced. The peaks for all three orders were visibly lower and more fragmented compared to the control group. Furthermore, venom presence induced a shift in successional kinetics: the *C. cerastes* group showed more rapid transitions between early colonizers, but with significantly lower carrying capacities for larval and adult populations. This visualization confirms that snake venom acts as a biological modifier that alters the magnitude of insect colonization (abundance) and the speed of the stages (kinetics) without fundamentally disrupting the ecological order of insect succession (sequence).

### 3.6. Correlation Between Insect Abundance and Environmental Factors

**Figure 8:**
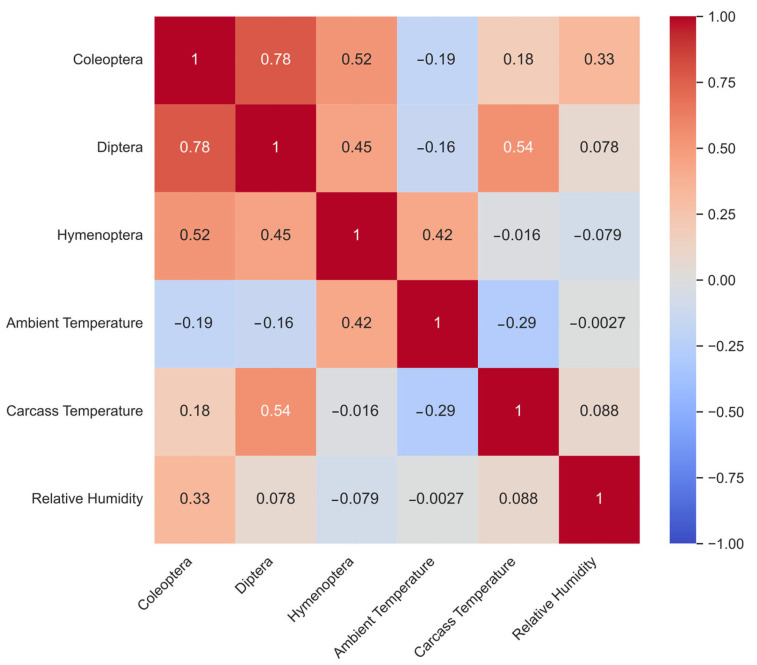
Spearman rank correlation coefficients were calculated to assess the relationships between insect orders (Coleoptera, Diptera, and Hymenoptera) and environmental variables, including ambient temperature, carcass temperature, and relative humidity, throughout carcass decomposition. The color gradient represents the strength of the correlations: red indicates a positive correlation, blue indicates a negative correlation, and the intensity of the color is proportional to the magnitude of the correlation coefficient.

A Spearman rank correlation analysis was conducted to evaluate the relationships between the abundance of the primary insect orders and key environmental variables, including ambient temperature, carcass temperature, and relative humidity (Figure 8). Strong positive correlations were observed among the three insect orders, indicating overlapping successional waves and concurrent colonization periods. The strongest correlation occurred between Coleoptera and Diptera (r_s_ = 0.78), suggesting that beetle and fly populations expanded and declined in close temporal proximity. Hymenoptera also exhibited moderate positive correlations with Coleoptera (r_s_ = 0.52) and Diptera (r_s_ = 0.45). The analysis further identified distinct thermal drivers for each insect order. Diptera displayed a moderate positive correlation with carcass temperature (r_s_ = 0.54), likely reflecting the heat generated by larval aggregations during the bloated and decay stages, which elevates internal carcass temperature above ambient levels. Hymenoptera showed a moderate positive correlation with ambient temperature (r_s_ = 0.42), indicating that foraging and predatory activities of ants were influenced more by external environmental warmth than by internal carcass heat. In contrast, ambient temperature exhibited weak negative correlations with Coleoptera (r_s_ = −0.19) and Diptera (r_s_ = −0.16), suggesting that periods of extreme external heat did not coincide with peak activity for these orders.

Relative humidity exhibited variable effects on the insect community. Coleoptera demonstrated the strongest association with this factor, showing a moderate positive correlation (r_s_ = 0.33). Diptera displayed a negligible positive correlation with humidity (r_s_ = 0.078), whereas Hymenoptera exhibited a weak negative correlation (r_s_ = −0.079). These results suggest that slightly drier conditions may favor Hymenopteran activity, while more humid conditions correspond to higher beetle abundance. Environmental factors themselves showed limited interdependence. A weak negative correlation was observed between ambient temperature and carcass temperature (r_s_ = −0.29), supporting the observation that carcass heating during active decomposition is driven primarily by larval metabolic activity rather than external weather conditions. Relative humidity remained largely independent of both ambient temperature (r_s_ = −0.0027) and carcass temperature (r_s_ = 0.088).

## 4. Discussion

A central question in forensic entomology is whether variations in carrion decomposition and arthropod succession are influenced solely by environmental conditions or by the cause of death itself. The present study demonstrates that snake envenomation constitutes an independent biological factor capable of accelerating decomposition and altering arthropod succession pattern, even under comparable environmental conditions. These findings address a critical gap in forensic entomology, where cause-of-death–specific effects remain underrepresented in succession-based PMI models [25,35,36,37].

One of the most consistent observations was the marked shortening of the fresh and bloated stages in envenomed carcasses. This acceleration cannot be attributed solely to abiotic factors, as temperature, humidity, and wind speed were comparable across treatments. Rather, it is most likely explained by venom-mediated enhancement of autolytic and microbial processes. Snake venoms contain proteolytic enzymes, phospholipase A2, and—in viperid species such as *C. cerastes*—high concentrations of metalloproteinases that induce rapid tissue necrosis [23,38,39]. These components initiate cellular breakdown before death, effectively preconditioning tissues for rapid postmortem degradation. Various local tissue alterations accompanying snake bite such as hemorrhage, edema, and myonecrosis may result in tissue loss or organs dysfunctions [23]. The more pronounced effects observed in *C. cerastes* compared to *N. haje* are consistent with the predominantly cytotoxic and hemorrhagic properties of viperid venoms, in contrast to the primarily neurotoxic profile of elapid venoms [17,24,38,40].

A central concern in forensic entomology is whether biological interference disrupts the fundamental pattern of insect succession. Our results indicate that snake envenomation modifies the rate of decomposition rather than the sequence of arthropod succession. Diptera remained the primary colonizers during the early decomposition stages across all treatments, confirming their ecological dominance and forensic relevance [25,35,41,42,43]. However, envenomed carcasses exhibited delayed oviposition, reduced larval aggregation, and lower overall larval abundance. This apparent paradox—accelerated decomposition alongside reduced dipteran larval density—suggests that, although venom-induced tissue degradation enhances early odor cues attractive to flies, residual venom components exert sublethal toxic or deterrent effects on developing larvae [25,35,36,42,44]. These findings highlight the need for caution when applying dipteran-based PMI models in envenomation cases, as neglecting venom effects may result in systematic overestimation of PMI [25,39,41].

Coleoptera exhibited a delayed but stable association with carcasses, with abundance increasing during the decay and dry stages, regardless of treatment. Their comparatively limited response to venom likely reflects an ecological reliance on desiccated tissues, larval remains, and secondary carrion resources rather than on fresh soft tissues [25,35,39]. This stability reinforces the utility of beetles as reliable indicators for late-stage PMI estimation, even under biologically modified decomposition conditions, and supports the notion that coleopteran succession is relatively resilient to chemical perturbations occurring in early decomposition stages [25,35,39,45].

Hymenoptera, particularly ants, represent an important but often underestimated component of venom-influenced decomposition dynamics. Although they are not primary PMI indicators, their early and abundant activity indirectly affects successional outcomes. Ants were especially active during the fresh and bloated stages on envenomed carcasses, exploiting exposed tissues and preying on dipteran eggs and early instar larvae. Their activity appeared more closely associated with surface tissue conditions and ambient environmental cues than with internal carcass temperature, which may explain their resilience to venom-modified microenvironments [35,36,39,45,46,47].

A comparative, multi-taxon evaluation revealed distinct order-specific responses to snake envenomation. Diptera exhibited altered developmental timing and reduced larval success. Hymenoptera showed increased early-stage abundance and enhanced trophic interactions. Coleoptera demonstrated stable late-stage colonization. This study focused on ordinal-level responses rather than family- or species-level dynamics. This approach was intentionally selected to capture broad ecological patterns relevant to forensic applications, which remain consistent despite variability at the species level. Future studies incorporating family- and species-level analyses may further refine PMI estimation models [25,35,37,39,44,45]. Correlation analyses further supported these distinctions. Dipteran abundance was positively associated with carcass temperature generated by larval mass, hymenopteran activity correlated with ambient temperature, and coleopteran presence exhibited a weaker dependence on thermal conditions. These patterns indicate that venom exposure functions as a biological modifier within environmental contexts, rather than operating independently of them [25,35,37,39,44,45].

From a forensic perspective, these findings carry significant implications. Applying standard entomological models to snakebite-related deaths without accounting for venom-induced acceleration of decomposition may systematically overestimate the PMI. Integrating observations of accelerated early decomposition, reduced dipteran larval density, increased hymenopteran activity, and sustained coleopteran presence reveals a distinctive successional pattern characteristic of envenomation cases. These results support recent recommendations for integrative, multi-order approaches in forensic entomology, particularly in complex deaths involving toxins or biologically active compounds [25,35,36,37,39,44].

Several limitations should be acknowledged. While enhancing generalizability, future studies incorporating species-specific developmental models and quantitative venom residue analyses may further refine PMI estimates under venom-related conditions [35,36,37,39,45]. Nonetheless, these findings provide a robust ecological framework for understanding how snake envenomation modifies carrion decomposition and arthropod succession. Another limitation to consider in future research is the use of an animal model more closely comparable to humans, such as pigs, when feasible, since there is broad consensus in the scientific community that data obtained from rabbits may not be directly applicable to humans [48,49].

In summary, snake envenomation can modify carrion ecology that accelerates early decomposition and elicits predictable, order-specific arthropod responses. Although the general successional sequence remains intact, venom exposure introduces measurable deviations in timing and abundance that are forensically significant. Incorporating cause-of-death–specific and multi-taxon perspectives into forensic entomology is therefore essential for improving PMI accuracy and advancing ecologically informed interpretations of insect evidence [25,35,36,37,39,44,45].

## 5. Conclusions

The present study demonstrates that snake envenomation constitutes a biologically significant modifier of carrion decomposition and insect succession, with direct implications for forensic PMI estimation. Envenomation with both *N. haje* and *C. cerastes* venoms significantly altered decomposition rates and patterns of insect colonization compared to non-envenomed controls, despite exposure to comparable environmental conditions. These results indicate that the cause of death, specifically venom-induced pathology, can independently influence postmortem ecological processes and should be considered in forensic analyses.

Envenomed carcasses exhibited an accelerated progression through the fresh and bloated stages, most pronounced in *C. cerastes*-treated carcasses, reflecting the potent cytotoxic and proteolytic activity of viperid venoms. In contrast, *N. haje* venom produced intermediate effects, consistent with its predominantly neurotoxic profile. Importantly, although the overall sequence of insect succession remained intact, venom exposure induced measurable shifts in the timing, abundance, and dominance patterns among arthropod orders.

Diptera remained the primary colonizers; however, overall abundance and persistence were reduced on envenomed carcasses, raising the potential for PMI overestimation if venom effects are ignored. Coleoptera exhibited comparatively stable late-stage colonization, reinforcing their reliability as indicators for advanced decomposition stages, whereas Hymenoptera played a prominent early role, likely mediating indirect effects through predation and competition.

Statistical modeling confirmed that insect abundance was jointly influenced by time, decomposition stage, insect order, and venom treatment, highlighting the complex, multifactorial nature of carrion ecology. Correlation analyses further revealed order-specific relationships with temperature and humidity variables, supporting the interpretation that venom exposure functions as a biological modifier within existing environmental frameworks rather than replacing them.

Collectively, these results demonstrate that snake envenomation induces predictable, order-specific deviations in carrion decomposition and arthropod succession. Standard entomological PMI models, when applied without accounting for venom-induced effects, are susceptible to systematic bias. Incorporating cause-of-death–specific modifiers and adopting a multi-taxon analytical approach can substantially improve PMI accuracy in envenomation-related cases. This study provides a preliminary experimental framework and baseline data for integrating venom effects into forensic entomology and underscores the need for future research involving species-level analyses, venom residue quantification, and larger animal models to further refine PMI estimation under toxin-mediated conditions.

## Figures and Tables

**Table 1:** Insect families and species (Diptera, Coleoptera, and Hymenoptera) recorded at different decomposition stages of rabbit carcasses in control and snake venom-treated groups (*Naja haje* and *Cerastes cerastes*).

Order	Family	Species	Control				*N. haje*				*C. cerastes*			
			Fr	Bl	De	Dr	Fr	Bl	De	Dr	Fr	Bl	De	Dr
**DIPTERA**	Calliphoridae	*Calliphora vicina*	−	**+**	**+**	**+**	−	**+**	**+**	−	−	**+**	**+**	−
		*Chrysomya albiceps*	**+**	**+**	**+**	**+**	**+**	**+**	**+**	**+**	**+**	**+**	**+**	**+**
		*Chrysomya megacephala*	−	**+**	**+**	**+**	−	**+**	**+**	**+**	**+**	**+**	**+**	−
		*Chrysomya rufifacies*	−	**+**	**+**	**+**	−	**+**	**+**	**+**	**+**	**+**	**+**	−
		*Lucilia cuprina*	**+**	**+**	**+**	**+**	−	**+**	**+**	−	**+**	**+**	**+**	−
		*Lucilia sericata*	**+**	**+**	**+**	**+**	**+**	**+**	**+**	**+**	**+**	**+**	**+**	**+**
	Muscidae	*Musca domestica*	**+**	**+**	**+**	**+**	**+**	**+**	**+**	**+**	**+**	**+**	**+**	**+**
		*Musca sorbens*	**+**	**+**	**+**	−	**+**	**+**	**+**	**+**	**+**	**+**	**+**	**+**
		*Musca stabulans*	**+**	**+**	**+**	−	**+**	**+**	**+**	**+**	**+**	**+**	**+**	**+**
		*Stomoxys calcitrans*	**+**	**+**	**+**	−	**+**	**+**	−	−	**+**	**+**	−	−
		*Synthesiomyia nudiseta*	−	**+**	**+**	**+**	−	**+**	**+**	−	−	**+**	**+**	**+**
	Sarcophagidae	*Sarcophaga argyrostoma*	**+**	**+**	**+**	**+**	**+**	**+**	**+**	**+**	**+**	**+**	**+**	**+**
		*Sarcophaga dux*	−	**+**	**+**	−	**+**	**+**	**+**	**+**	**+**	**+**	**+**	**+**
		*Wohlfahrtia nuba*	−	**+**	**+**	**+**	−	**+**	**+**	**+**	**+**	**+**	**+**	−
	Drosophilidae	*Drosophila repleta*	−	**+**	**+**	−	−	**+**	**+**	−	−	**+**	**+**	−
	Fanniidae	*Fannia canicularis*	−	**+**	**+**	−	−	**+**	**+**	**+**	−	**+**	**+**	**+**
	Hippoboscidae	*Hippobosca equina*	−	−	**+**	−	−	−	**+**	**+**	−	−	**+**	−
	Phoridae	*Megaselia scalaris*	−	**+**	**+**	**+**	−	**+**	**+**	−	−	−	**+**	**+**
	Piophilidae	*Piophila casei*	−	−	**+**	−	−	−	**+**	−	−	−	**+**	**+**
	Sphaeroceridae	*Coproica vagans*	−	−	**+**	−	−	−	**+**	−	−	−	**+**	**+**
	Ulidiidae	*Physiphora alceae*	−	−	**+**	−	−	−	**+**	−	−	−	**+**	**+**
**COLEOPTERA**	Cleridae	*Necrobia rufipes*	−	**+**	**+**	**+**	−	**+**	**+**	**+**	−	**+**	**+**	**+**
	Dermestidae	*Attagenus* sp.	−	**+**	**+**	**+**	−	**+**	**+**	**+**	−	**+**	**+**	**+**
		*Dermestes frischii*	−	**+**	**+**	**+**	−	**+**	**+**	**+**	−	**+**	**+**	**+**
		*Dermestes maculatus*	−	**+**	**+**	**+**	−	**+**	**+**	**+**	−	**+**	**+**	**+**
	Staphylinidae	*Bledius* sp.	−	−	**+**	−	−	**+**	**+**	**+**	−	**+**	**+**	**+**
	Tenebrionidae	*Apentanodes* sp.	−	**+**	**+**	**+**	−	**+**	**+**	**+**	−	**+**	**+**	−
**HYMENOPTERA**	Formicidae	*Camponotus* sp.	**+**	**+**	**+**	**+**	**+**	**+**	**+**	**+**	**+**	**+**	**+**	−
		*Cataglyphis* sp.	**+**	**+**	**+**	**+**	**+**	**+**	**+**	**+**	**+**	**+**	**+**	−
		*Messor abeillei*	**+**	**+**	**+**	**+**	**+**	**+**	**+**	**+**	**+**	**+**	**+**	−

Fr: Fresh stage; Bl: Bloating stage; De: Decay stage; Dr: Dry stage. (+) Species recorded; (−) Species not recorded.

**Table 2:** Negative binomial regression analysis of insects’ abundance on rabbit carcasses under different venom treatments.

Parameter	Type II Tests (Overall Effects)			Model Coefficients			
	LR χ^2^	df	*p*-Value	β (Estimate)	SE	z	*p*-Value
Days	43.28	1	<0.001	−0.105	0.016	−6.677	<0.001
Treatment	15.15	2	<0.001				
*C. cerastes* (Ref)				–	–	–	–
*N. haje*				−0.009	0.060	−0.156	0.876
Control				0.206	0.068	3.043	0.002
Decomposition Stage	307.04	3	<0.001				
Bloated (Ref)				–	–	–	–
Decayed				0.275	0.095	2.899	0.004
Dried				−0.336	0.172	−1.955	0.051
Fresh				−1.333	0.107	−12.494	<0.001
Order	537.62	2	<0.001				
Coleoptera (Ref)				–	–	–	–
Diptera				1.371	0.061	22.418	<0.001
Hymenoptera				1.055	0.063	16.867	<0.001

SE = Standard Error; LR χ^2^ = Likelihood Ratio Chi-square.

## Data Availability

The original contributions presented in this study are included in the article. Further inquiries can be directed to the corresponding author.
